# Amyloid-beta antibody treatment in Alzheimer’s disease: An update on recent data and outlook on implementation in clinical routine

**DOI:** 10.1007/s00508-024-02466-7

**Published:** 2024-11-06

**Authors:** Elisabeth Stögmann, Reinhold Schmidt

**Affiliations:** 1 Department of Neurology, Medical University of Vienna, Waehringer Gürtel 18–20, 1090 Vienna, Austria; 2 Department of Neurology, Medical University of Graz, Auenbruggerplatz 22, 8036 Graz, Austria

**Keywords:** Alzheimer, Dementia, Monoclonal antibody, New therapeutic strategy, Use recommendation

## Abstract

Amyloid-beta (Aβ) antibody treatment has emerged as a promising approach for the treatment of Alzheimer’s disease (AD), targeting the accumulation of Aβ plaques, which are a hallmark of the disease. This review provides an update on recent clinical trial data, highlighting the efficacy and safety of various antibodies targeting Aβ. Recent trials have demonstrated that certain Aβ antibodies can reduce amyloid plaques and slow cognitive decline in patients with early AD. Key findings from trials of drugs are discussed, including their mechanisms of action, dosing regimens, and observed side effects. The potential for Aβ antibody therapy to be integrated into routine clinical practice is also explored. While Aβ antibody therapy represents a significant advancement in AD treatment, ongoing research is needed to optimize their use and understand their long-term impact. This review underscores the importance of personalized medicine in AD and the need for continued innovation in therapeutic strategies.

## Introduction

Alzheimer’s disease is one of the widespread diseases in aging societies. In Austria, around 150,000 people are currently affected. Pathological changes of Alzheimer’s disease can be detected in the brains of affected individuals 20 or more years before the onset of the first symptoms [1]. According to the amyloid cascade hypothesis and its extensions [2], amyloid proteinopathy represents the initial central event in the pathophysiology that interacts with tau proteinopathy [3] and then triggers a multitude of further molecular cascades leading to synaptic dysfunction and neurodegeneration culminating in cognitive decline and dementia [4].

Currently, the focus of interest in drug development is disease modification through monoclonal antibodies targeting different stages of the amyloidβ (Aβ) aggregation cascade. Monoclonal antibodies lead to Aβ removal from the brain by binding against specific epitopes of aggregated β‑amyloid, facilitating Aβ clearance from the brain. This process potentially mitigates both direct and downstream negative effects of Aβ, including tau pathology, and slowing of cognitive decline [5]. Recently, phase 3 trials demonstrated for the first time that monoclonal antibodies reduce amyloid deposits and thereby slow the progression of symptoms of the disease [6–11]. Our article summarizes the latest findings on amyloid‑β antibody therapy in Alzheimer’s disease and discusses possible implications for treatment decisions and patient management.

## Lecanemab—The first anti-amyloid antibody with consistent positive results on biomarker and clinical endpoints

In January 2023, the study results of lecanemab were published [7]. Lecanemab is a humanized monoclonal immunoglobulin gamma‑1 (IgG 1) antibody directed against soluble (protofibrils) and insoluble forms of amyloid beta (Aβ). Lecanemab was investigated in an 18-month, multicenter, randomized, double-blind, placebo-controlled phase 3 study (Clarity-AD). Included in the study were 1795 subjects aged 50–90 years with early stage Alzheimer’s disease (mild cognitive impairment or mild dementia due to Alzheimer’s disease) with evidence of amyloid‑β in amyloid-PET (positron emission tomography) or corresponding evidence of amyloid‑β pathology in cerebrospinal fluid (CSF). Participants were randomly assigned in a 1:1 ratio to receive intravenous lecanemab treatment or placebo every 2 weeks.

The primary endpoint was the clinical dementia rating-sum of boxes (CDR-SB), an integrated scale that assesses both cognitive and functional components. The CDR-SB assesses six domains considered important by patients and caregivers (memory, orientation, judgment, and problem solving, community affairs, home and hobbies, and personal care). The total score ranges from 0 to 18, with higher scores indicating more severe impairment. Lecanemab-treated patients showed on average less decline on the CDR-SB score than patients on placebo (1.21 versus 1.66, respectively; difference: −0.45; 95% confidence interval, CI: −0.67 to −0.23; *P* < 0.001). In relative terms, this difference corresponded to a 27% slowing of cognitive decline in the lecanemab group in comparison to placebo. The study also met almost all secondary endpoints, which included numerous cognitive and functional scales. The reduction in brain amyloid deposits in PET was significantly greater with lecanemab than with placebo. Other results such as cerebrospinal fluid and blood biomarkers indicated a similar trend. The slowing of decline in the CDR-SB as compared to placebo related to a 5.3-month delay in clinical progression over the observational period of 18 months.

**Fig. 1:**
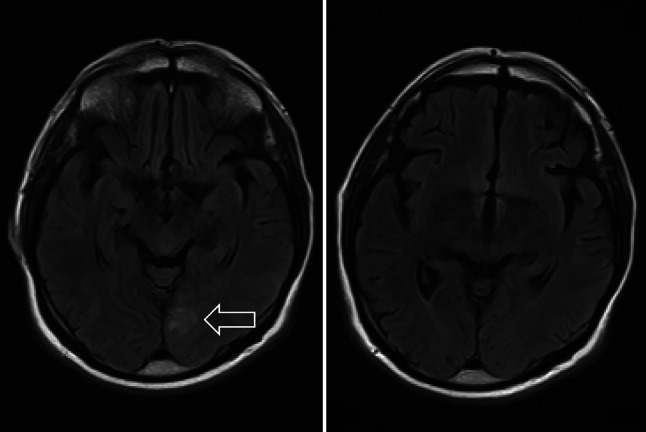
Amyloid-related imaging abnormality-edema (ARIA‑E; arrow) in a patient under administration of aducanumab, a monoclonal amyloid antibody which did not receive approval in Europe; shown is a Magnetic resonance imaging (MRI) with two axial sections, performed during the Phase 3 study Engage at the Medical University Graz. The left image shows ARIA - E in the left occipital lobe, the right image shows complete remission after discontinuation of the therapy within 1 month

As with other anti-amyloid‑β antibody therapies in the past, so-called amyloid-related imaging abnormalities with edema (ARIA‑E; Fig. 1) and microbleeds (ARIA-H) in the brain occurred significantly more frequently in the verum than in the placebo group. These changes were identified during scheduled magnetic resonance imaging (MRI) follow-up investigations. The acronym ARIA is a general term, which covers 2 classes of MRI signal alterations: ARIA‑E (edema) refers to parenchymal edema and sulcal effusion, which commonly manifest as transient hyperintensities on fluid-attenuated inversion recovery or T2-weighted MRI sequences, with no restricted diffusion abnormalities and ARIA‑H (hemorrhage) refers to deposits of hemosiderin (i.e., a blood degradation product), including parenchymal microhemorrhages (< 10 mm or < 5 mm according to different studies) and leptomeningeal superficial siderosis. The ARIA‑H manifests as very low-intensity signals, detected on gradient echo or susceptibility-weighted imaging MR sequences. The ARIA‑E and ARIA‑H are thought to be expressions of an increased vascular fragility and leakage of proteinaceous fluid and erythrocytes caused by the therapeutic effect of monoclonal antibodies [12].

Apart from ARIA‑E (12.6% with lecanemab and 1.7% with placebo) and ARIA‑H (17.3% with lecanemab and 9% with placebo), infusion-related reactions were the most relevant and frequent adverse events (26.4% in the lecanemab group and 7.4% in the placebo group). A detailed description of frequency of ARIA is shown in Table 1. The incidence of symptomatic ARIA‑E was 2.8% and that of symptomatic ARIA‑H was 0.7%. The most frequently reported symptoms related to occurrence of ARIA were headaches, visual disturbances and confusion. Most of ARIA occurred in the first 6 months of the treatment period (92%) and disappeared within 4 months of detection (81%). The incidence of ARIA‑E was higher in Apolipoprotein (*APOE*) *ε4* carriers compared to non-carriers (15.8% vs. 5.4%), with the incidence of ARIA‑E being 32.6% in homozygous carriers and 10.9% in heterozygous carriers. The occurrence of any serious adverse event due to ARIA (E or H) was 0.6–0.8%. During the double-blind phase of the lecanemab studies no participant died because of the occurrence of ARIA; however, three deaths were reported in the open-label extension study, which were possibly related to ARIA.

**Table 1:** ARIA‑E and ARIA‑H occurrence in patients treated by lecanemab or donanemab

	Lecanemab (%)			Donanemab (%)		
	All participants	*APOE ε4* heterozygotes	*APOE ε4* homozygotes	All participants	*APOE ε4* heterozygotes	*APOE ε4* homozygotes
ARIA‑E	12.6	10.9	32.6	24	22.8	40.6
Sympt. ARIA‑E	2.8	1.7	9.2	6.1	NA	NA
ARIA‑H all treatment emergent	17.3	14	39	19.7	NA	NA
ARIA‑H microbleeds	14	12.1	34	NA	–	–
ARIA‑H superf. siderosis	5.6	4.0	12.8	NA	–	–
Sympt ARIA‑H	0.7	1.0	0	1.0	–	–
Any SAE due to ARIA	ARIA‑E: 0.8: ARIA-H: 0.6	ARIA-E:0.4 ARIA-H:0.2	ARIA-E:2.1 ARIA-H: 1.4	ARIA-E:1.5 ARIA-H:0.5	–	–
Macrohemorrhage or ICB >1 cm	0.7	0.6	1.4	0.4	–	–

*ARIA* amyloid-related imaging abnormalities, *ARIA‑E* amyloid-related imaging abnormalities with edema, *ARIA‑H* amyloid-related imaging abnormalities with hemorrhage, *Sympt.* symptomatic, *Superf. siderosis* superficial siderosis, *SAE* serious adverse event, *ICB* intracerebral bleeding

In January 2023 lecanemab received accelerated U.S. Food and Drug Administration (FDA) approval which was followed by full approval in July 2023 based on the clinical efficacy data. This was the first time an anti-amyloid‑β antibody has received full FDA approval. An application for approval was also put forward to the EMA, the final decision is currently pending and is expected in 2024.

## Donanemab data provide further evidence

In July 2023, data on the results of the donanemab study (Trailblazer-Alz 2) were published. The study design differed slightly from the other antibody studies [10].

Donanemab is a monoclonal immunoglobulin G1 antibody directed against an N‑terminal pyroglutamate epitope present in mature Aβ plaques. Trailblazer-Alz 2 was a 76-week, multicenter, randomized, double-blind, placebo-controlled phase 3 study. The study was originally planned as a phase 2 study but was converted to a larger phase 3 study in February 2021 to confirm and expand on the results of the earlier Trailblazer-Alz study [11]. It included 1736 participants aged 60–85 years with early symptomatic Alzheimer’s disease (mild cognitive impairment or mild Alzheimer’s disease). Eligible participants were randomized in a 1:1 ratio to receive donanemab or placebo and the substance was administered intravenously every four weeks. Eligible participants had mini-mental state examination (MMSE) screening scores ranging from 20 to 28, amyloid pathology (≥ 37 centiloids) assessed by 18F-florbetapir13 or 18F-florbetaben14 positron emission tomography (PET), and tau pathology assessed through 18F-flortaucipir PET imaging with centralized image interpretation. Centiloids are a unit of measurement for the amyloid burden in the brain of Alzheimer’s patients, with a value below 30 considered negative or normal.

Before randomization patients were divided into groups with low/medium tau burden (68.1% of participants) or high tau burden (31.8%) based on baseline tau PET values. If the amyloid plaque concentration (measured after 24 and 52 weeks) on a single amyloid PET scan was less than 11 centiloids or less than 25 but greater than or equal to 11 centiloids on two consecutive PET scans, donanemab was switched blindly to placebo.

Unlike other studies, the primary endpoint was the integrated Alzheimer’s disease rating scale (iADRS). The iADRS is an integrated assessment of cognitive abilities and daily functioning based on the 13-item cognitive subscale of the Alzheimer disease assessment scale (ADAS-Cog13) and the Alzheimer disease cooperative study-instrumental activities of daily living (ADCS-iADL). It measures the severity of Alzheimer’s disease in a single summary score. Possible scores on the iADRS range from 0 to 144 (lower scores indicate greater impairment). In the low-medium tau population, the change in iADRS score from baseline after 76 weeks was −6.02 (95% CI −7.01 to −5.03) in the donanemab group and −9.27 (95% CI −10.23 to −8.31) in the placebo group (difference 3.25, 95% CI 1.88–4.62; *P* < 0.001), corresponding to a 35.1% slowing of disease progression. In the overall population (low-medium tau population + high tau population), the change in iADRS score from baseline after 76 weeks was −10.19 (95% CI −11.22 to −9.16) in the donanemab group and −13.11 (95% CI −14.10 to −12.13) in the placebo group (difference, 2.92, 95% CI 1.51–4.33; *P* < 0.001), corresponding to a 22.3% slowing of disease progression in the verum group versus placebo. For the CDR-SB in the low-medium tau population, the difference of the change between the treatment groups after 76 weeks was −0.67 (95% CI, −0.95 to −0.40), indicating 36.0% slowing of clinical progression. In the combined population, the differences in CDR-SB between the donanemab and placebo groups were −0.70 (95% CI −0.95 to −0.45), indicating a 28.9% slowing of clinical progression. Similar treatment benefits were seen for all secondary clinical endpoints, with low-middle tau patients generally showing larger effect sizes than those in the overall population. This result is in line with the view that treatment in earlier disease stages is more efficient as compared to treatment at more advanced stages of Alzheimer’s disease. After 76 weeks disease progression was delayed by 4.4 months on the iADRS and by 7.5 months on the CDR-SB in the low-moderate tau group under donanemab treatment. The percentages of participants treated with donanemab who achieved amyloid clearance to a normal level at 76 weeks was 80.1% in the low-medium tau population and 76.4% in the combined population. The effects of treatment on biomarkers of Alzheimer’s disease downstream of Aβ were variable. Donanemab led to a significant reduction in plasma P‑tau217 concentrations, a marker of Aβ-mediated tau phosphorylation and secretion; however, no effect was observed on longitudinal tau PET in the frontal cortex region.

During the Trailblazer-Alz 2 study, 3 participants in the donanemab group died with severe ARIA (2 heterozygous *APOE ε4* carriers and one non-carrier; none were prescribed anticoagulants or antiplatelet agents; one participant resumed treatment after resolution of severe ARIA‑E and severe ARIA‑H, and one participant had superficial siderosis at baseline). The ARIA‑E occurred in 24.0% of the donanemab group, with most being mild to moderate; 6.1% experienced symptomatic ARIA‑E, a detailed description is shown in Table 1. Most cases of first ARIA‑E (57.9%) occurred after approximately 3 donanemab infusions. The ARIA‑E was numerically less frequent in *APOE ε4* non-carriers compared to carriers, with a higher frequency in homozygotes (40.6%) than in heterozygotes (22.8%), compared to *APOE ε4* non-carriers (15.7%). The occurrence of any serious adverse event due to ARIA (E or H) was 0.5–1.5%. Infusion-related reactions were reported in 8.7% of participants in the donanemab group.

The design of Trailblazer-Alz 2 included two innovative aspects that could impact patient care. Firstly, the amyloid plaque burden was assessed by PET at 24 and 52 weeks, and patients who met predefined discontinuation thresholds in PET were switched from donanemab to placebo, which occurred in 52% of patients in the treatment group. Considering the high patient burden and expected costs of Aβ-directed monoclonal antibodies, a limited treatment duration could significantly improve the feasibility of the treatment. Secondly, patients were stratified based on the baseline tau PET, with patients in the low-medium tau group benefiting more, while patients in the high tau group showed little to no clinical benefit compared to placebo. These results suggest that in addition to clinical criteria and Aβ biomarker positivity, the classification of tau may be critical in identifying patients who would benefit the most; however, the implementation of these techniques in clinical practice will be challenging given the limited access to Aβ-PET and especially tau-PET. Further research is needed to determine whether blood-based biomarkers could replace expensive PET scans in measuring biomarker treatment response and in predicting clinical response in the future.

In July 2024 donanemab received full FDA approval. An application for approval was also put forward to the EMA, the final decision is currently pending and is expected in 2025.

## Scepticism about varying clinical efficacy of amyloid-β antibodies in Alzheimer’s disease

Over the years, there have been many contradictory and negative clinical results in the research on Alzheimer’s therapy regarding anti-amyloid‑β antibodies, which have left skepticism about this therapeutic concept to this day. At least two amyloid‑β antibodies, lecanemab and donanemab, have recently shown clear and consistent positive results, not only regarding biomarkers of Alzheimer’s disease but also in terms of clinical benefits. This marks a milestone in Alzheimer’s therapy research.

The reason for the discrepant study results of the amyloid‑β antibodies has not yet been clarified. It is suspected that different biological properties of the antibodies and differences in the study design played a role. Table 2 lists the study results of recent antibody studies and compares the extent of amyloid reduction at the end of the study period (measured in centiloids) with the clinical outcome, based on the CDR-SB. The results of previously reported monoclonal amyloid antibodies, such as aducanumab and gantenerumab are also shown in Table 2, although these showed no proven clinical effects.

**Table 2:** Overview of specific study results of different monoclonal amyloid antibodies

*Trial positive*	*Antibody*	*Duration (years)*	*Baseline amyloid (centiloids)*	*Residual amyloid (centiloids)*	*CDR-SB delta vs. placebo (%)*
Emerge	Aducanumab	1.5	85	25	−22
Study 201 (Ph2)	Lecanemab	1.5	75	6	−26
Clarity AD	Lecanemab	1.5	78	23	−27
TrailblazerAlz (Ph2)	Donanemab	1.5	108	23	−23
TrailblazerAlz 2^*^	Donanemab	1.5	102	14	−36
*Trial negative*	*Antibody*	*Duration (years)*	*Baseline amyloid (centiloids)*	*Residual amyloid (centiloids)*	*CDR-SB delta vs. placebo (%)*
Engage	Aducanumab	1.5	91	37	2
Graduate I	Gantenerumab	2.25	92	34	−8
Graduate II	Gantenerumab	2.25	98	51	−6

Centiloids are a unit of measurement for the amyloid burden in the brain of Alzheimer’s patients, with a value below 30 considered negative or normal. CDR-SB (Clinical dementia rating-sum of boxes) is a neuropsychological test, further described in the text. Some data on previously reported trials relevant for this overview (Study 201 Lecanemab [8], Emerge/Engage trial with aducanumab [6], Graduate I + II with gantenerumab [11]) are also shown in the Table 2.

*Data shown for low and medium tau populations according to Tau-PET analysis of patients

One possible interpretation of these study results, currently discussed in the field of dementia research, is that the clinical effectiveness of an antibody directed against amyloid‑β may indeed depend on the extent of amyloid‑β reduction in the brain. According to this view, a reduction of amyloid‑β to the normal range would be necessary to achieve clinically significant results. This interpretation represents the view of many experts in the field but must currently be considered as preliminary due to the insufficient availability of data. Table 2 shows that all antibody studies demonstrating clinically significant results have lowered the centiloid value below the relevant threshold of 30.

## Recommendation for appropriate use

Despite these significant advances, there is still much to learn about the different mechanisms of action of the new therapies. The positive effects of lecanemab and donanemab must be weighed against their risks. In this context, various authors have published therapy recommendations [13, 14]. Patients eligible for these therapies will have to meet the clinical criteria for mild cognitive impairment due to Alzheimer’s disease or mild Alzheimer’s dementia and show biomarker evidence of underlying amyloid pathology (amyloid PET or CSF). In the future, plasma biomarkers may be useful for screening patients, however, it is unlikely that plasma biomarkers will completely replace CSF biomarkers or amyloid PET. When ARIA occur they are usually asymptomatic and in the majority of cases resolve within an approximate time of 10 weeks. If symptoms occur they are usually mild and consist of headaches or confusion but they can also include more severe symptoms such as epileptic seizures. In some cases, these events can be life-threatening and in rare cases can lead to death. It will be important to consider risk mitigation strategies. Before starting therapy, an MRI needs to be performed to exclude patients with evidence of significant cerebral amyloid angiopathy (CAA). Conducting regular MRI examinations during treatment and incorporating conservative algorithms for interrupting or discontinuing treatment if ARIA occurs must be ensured. A total of five MRI scans are recommended during the first year of treatment. An MRI scan should be performed before the implementation of therapy and during the whole period of treatment, especially in *APOE ε4* carriers or in patients with previous ARIA events. Sensitivity to clinical symptoms associated with ARIA and neuroradiology expertise will need to develop. It will be critical to consider that patients receiving anticoagulation are probably at higher risk for ARIA‑H and associated complications. For now, patients receiving anticoagulation will not be eligible for therapy with monoclonal amyloid‑β antibodies. Treatment with acute thrombolytics is also not recommended at this point. Expert panels recommend conducting *APOE* genotyping and discussing the patients’ *APOE* status and their risk of developing ARIA. Determining *APOE* status is important considering the higher risk of ARIA in *APOE ε4* carriers, especially in homozygous carriers. Counselling on *APOE* genotype will require more knowledge, time, and effort than before, even for physicians with experience in Alzheimer’s therapy. Patient-centered discussion with shared decision-making before initiating treatment is recommended. A registry needs to be established to track long-term clinical outcomes and adverse events in patients treated with monoclonal Aβ antibodies. Internationally, the inclusion of patients in the Alzheimer’s Treatment and Diagnostics Network (ALZ-NET; www. alz-net.org) or similar registries is recommended. Understanding the long-term efficacy and safety profile of these therapies under real-world conditions will be crucial for successful integration into clinical practice. Finally, it needs to be emphasized that clinical trial data regarding the use of these antibodies in certain populations, including underrepresented minority groups, as well as patients with autosomal dominant Alzheimer’s disease and patients with Down syndrome, have limitations. An important question in clinical practice and for health insurance companies is whether and when treatment can be discontinued in the context of progressive dementia or increasing frailty; however, these questions can only be answered once data on long-term treatment are available.

## Perspective of new therapeutic developments in AD therapy

There is currently a significant discrepancy between the number of patients expected based on demographic considerations and the number of patients currently treated in existing specialized outpatient clinics who would be eligible for treatment according to the inclusion criteria of the lecanemab and donanemab studies. The reasons for this are complex. Social stigma on the part of patients and the lack of availability of a causal therapy for Alzheimer’s disease on the part of healthcare professionals have certainly been reasons for low early diagnosis rates of dementia in our healthcare system in recent years [15]. Patients often seek specialized medical evaluation only in later stages of the disease, stages where disease-modifying therapy will no longer be applicable. Furthermore, the diagnosis with biomarkers (amyloid PET or AD biomarkers in cerebrospinal fluid) required for such a therapy is very resource-intensive and therefore not widespread. In addition, from a specialist’s perspective, many patients would be excluded from anti-amyloid‑β antibody therapy due to the currently known spectrum of side effects (patients with microbleeds and/or numerous microangiopathic lesions, patients on anticoagulant therapy). These points are highlighted in this issue in the manuscript “Monoclonal anti-amyloid antibody therapy: the epidemiological profile of target patients in Austria and the status of therapy-eligible patients registered at the memory clinic” by Lee et al.

Effective treatment of AD is a major unmet medical need. Slowing the progression of cognitive and functional decline with stabilizing impact on quality of life of patients and care givers by directly impacting on the underlying pathology is a fundamental step forward. At the present time it appears to be confirmed that monoclonal antibodies affect various stages of the amyloid‑β cascade, reduce amyloid‑β pathology via microglia-mediated activity, and can also reduce tau pathology and other neurodegenerative processes ultimately leading to clinical improvement. Beyond the individual patient’s perspective, we are currently experiencing the beginning of a new era of treatment of neurodegenerative diseases, transitioning from symptomatic to causal interventions. There is uncertainty about the actual extent and clinical meaningfulness of the clinical effect of these treatments but also about the proposed increase in the clinical effects when treatment is applied beyond a time of 18 months. It is also unclear if the treatment can be given intermittently, once the amyloid load is lowered to the threshold of normality. These open questions can only be addressed by long-term data collection in the context of ongoing studies. There are also studies which are investigating whether the clinical efficacy of monoclonal antibodies directed against Aβ is higher in even preclinical stages of Alzheimer’s disease.
